# An “*s*‐Electron” Donor Band Driven Metallic Ferromagnetism in Co‐Doped ZnO Films

**DOI:** 10.1002/advs.202508148

**Published:** 2025-07-26

**Authors:** Pei‐Yu Chuang, Jung‐Chun‐Andrew Huang, Ashish Atma Chainani, Hua‐Shu Hsu, Yen‐Fa Liao, Chang‐Yi Sung, Chih‐Hua Liu, Chien‐Yu Liao, Chi‐Hsuan Lee, Ku‐Ding Tsuei

**Affiliations:** ^1^ Department of Physics National Cheng Kung University Tainan 701 Taiwan; ^2^ National Synchrotron Radiation Research Center Hsinchu 300 Taiwan; ^3^ Department of Applied Physics National University of Kaohsiung Kaohsiung 81100 Taiwan; ^4^ Taiwan Consortium of Crystalline Materials (TCECM) National Science and Technology Council Taipei 106 Taiwan; ^5^ Department of Applied Physics National Ping Tung University Pingtung 90003 Taiwan; ^6^ Research Center for Applied Sciences Academia Sinica Taipei 11529 Taiwan

**Keywords:** Co‐doped ZnO, dilute magnetic semiconductor, donor electrons, ferromagnetism, HAXPES, impurity band

## Abstract

Achieving room‐temperature ferromagnetism (RTFM) in diluted magnetic semiconductors (DMSs) has been a long‐standing challenge, with doping transition metals (TM) into oxide semiconductors being one of the most common approaches. However, the underlying physical mechanisms remain poorly understood, particularly for Co‐doped ZnO (Co:ZnO) films, which exhibit high Curie temperatures (T*c*) above 300 K. A promising mechanism proposed for high‐T*c* ferromagnetism is the donor impurity band exchange model, in which donor electrons mediate the coupling between TM spins. Despite its theoretical significance, the nature of the donor band electrons has yet to be experimentally identified. In this work, we use polarization‐dependent, bulk‐sensitive hard x‐ray photoemission spectroscopy (HAXPES) to investigate Co‐doped ZnO epitaxial films. Our results reveal the presence of a weak electron donor band, crossing the Fermi level, and from a polarization dependence analysis, it is unambiguously identify it as having “*s‐*character.” This finding offers new insight into the ferromagnetic mechanism in Co‐doped ZnO, where Zn^1+^4*s* ^1^ states mediate the ferromagnetism, contributing to metallic‐like transport and Co^2+^ spin ordering. These results not only elucidate the complementary role of dopant‐host electronic states but also open avenues for designing novel room‐temperature magnetic semiconductors, particularly in the context of 2D DMSs.

## Graphical abstract

Hard X‐ray photoemission spectroscopy with polarization dependence enables orbital‐selective probing of buried electronic states. By tuning the incident light's polarization vector, symmetry‐resolved spectral features of core and valence states are revealed, offering deeper insights into orbital hybridization and spin–orbit interactions in functional oxide systems.

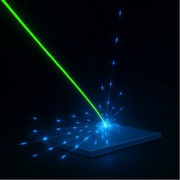

## 1 Introduction

Diluted magnetic semiconductors (DMSs) possess a combination of semiconducting and ferromagnetic properties, holding great potential for spintronic applications.^[^ ^1^, ^2^ ^]^ Early research focused on III‐V DMSs,^[^ ^3^ ^]^ in which the carrier‐mediated mechanism was established as the primary driver of ferromagnetism.^[^ ^4^ ^]^ However, the observed Curie temperatures (T*_c_*) observed in these materials remain well below room temperature, greatly restricting their applications. On the other hand, transition metal (TM) doped oxides (DMOs) such as Co‐doped ZnO have emerged as key DMS materials due to their potential for high Curie temperatures above room temperature, which is crucial for practical applications. Despite extensive studies on these materials, the origin of ferromagnetism in DMOs remains a subject of considerable debate.^[^ ^5^, ^6^, ^7^, ^8^, ^9^, ^10^ ^]^ Several mechanisms have been proposed to explain the ferromagnetism observed in DMO systems, with the donor impurity band exchange model being particularly promising. This model posits that donor electrons form a spin‐split band that mediates the coupling between TM spins, explaining the ferromagnetic behavior.^[^ ^11^ ^]^ However, to date, the experimental identification of the donor band has been elusive, preventing a direct connection between electronic structure and magnetic behavior. Advancing our understanding of room‐temperature DMOs requires experimental protocols/designs that uncover key physical mechanisms. Such insights could enable the development of novel room‐temperature magnetic semiconductors, including 2D DMSs.^[^ ^12^, ^13^, ^14^, ^15^, ^16^, ^17^, ^18^, ^19^ ^]^

DMOs are typically n‐type semiconductors, with doping concentration x below 5%, insufficient for magnetic percolation. The magnetic moment per TM in DMOs is proportional to x or x^1/2^ within the dilute limit, but diminishes as x increases beyond a threshold value.^[^ ^10^ ^]^ According to mean field theory results, RTFM in DMOs is not expected, and yet experimental observations contradict this prediction.^[^ ^20^ ^]^ Extensive studies have been TM substitution for host cations and ruled out extrinsic magnetic contribution from TM clusters.^[^ ^21^ ^]^ However, structural defects, such as oxygen vacancies and zinc interstitials, are known to play a crucial role in achieving RTFM in DMOs.^[^ ^22^, ^23^, ^24^, ^25^ ^]^

The donor impurity band exchange mechanism, proposed by Coey et al.,^[^ ^11^ ^]^ suggests that hybridization between TM states and defects influences the magnetic and electrical properties of DMOs. In ZnO‐based DMOs, impurity bands arising from defects or TM 3*d* states can either behave as localized electrons and retain semiconducting behavior, or broaden with increased defect density, enabling metallic transport.^[^ ^26^ ^]^

Experimental evidence highlights the complexity of ferromagnetism in DMOs. While Co *L*‐edge X‐ray magnetic circular dichroism (XMCD) confirms ferromagnetic signals in Co:ZnO, Co *M*‐edge resonant photoemission indicates negligible Co 3*d* states near the Fermi level (*E_F_*), contradicting the impurity band hypothesis.^[^ ^27^ ^]^ Additionally, electron‐induced MCD (EMCD) confirms the intrinsic ferromagnetism of Co^2+^ in ZnO nanoparticles.^[^ ^28^ ^]^ Interestingly, weak *d*⁰ ferromagnetism, observed in defect‐rich oxides without TM doping, further complicates the debate.^[^ ^29^ ^]^ Two key challenges hinder direct validation of the impurity‐band exchange model: 1) structural defects in DMOs are sensitive to ambient conditions, affecting reproducibility, and 2) existing measurement techniques, such as XMCD and EMCD, are either surface‐sensitive or limited to nanoscale regions, making them inadequate for bulk electronic characterization.^[^ ^20^, ^28^ ^]^

In this study, we address these challenges by developing fabrication methods that preserve structural defects and employing polarization‐dependent hard X‐ray photoemission spectroscopy (HAXPES) to probe the orbital character of bulk electronic structure. Our findings provide direct evidence of “*s*‐character” electron‐doping‐induced metallic ferromagnetism in Co‐doped ZnO, offering crucial insights into the electronic origins of RTFM in DMOs. These results pave the way for advancements in dilute magnetic semiconductors.

Epitaxial Co‐doped ZnO films were grown using radio frequency magnetron sputtering (RF sputtering) on sapphire substrates, maintaining a low Co doping concentration (≈5 at%) to avoid percolation effects. The films were carefully optimized to preserve structural defects, such as zinc interstitials and oxygen vacancies, which are known to play a significant role in magnetic behavior. The polarization dependence of the HAXPES spectra allowed us to probe the orbital character of the electronic states near *E_F_*. The bulk sensitivity of HAXPES provided key insights into the electronic structure that are not affected by surface contamination or degradation, thus eliminating problems encountered in soft X‐ray techniques such as XMCD or EMCD.

In order to carry out a careful comparative analysis, four sets of samples were prepared on Al_2_O_3_ (0001) substrates by RF‐sputtering in this study. The concentration of oxygen vacancies was controlled by varying the H_2_ content mixed with Ar during deposition. Sample A and B contained 5 at% Co doping in a 40 nm Co:ZnO layer. The H_2_/Ar ratios during sputtering were 5% for sample A and 2.5% for sample B. After deposition, both samples were capped with a 2 nm ZnO protective layer with no H_2_ during sputtering. Sample C was structurally identical to sample A but lacked the ZnO protective layer. These samples facilitate the investigation of the presence and absence of defects, such as oxygen vacancies, while eliminating charging effects during HAXPES measurements and thereby enable a reliable comparison of their relationship with variation in magnetic properties. Additionally, to compare the effects of Co doping on ZnO, a highly conductive ZnO film (40 nm) was also prepared, denoted as sample D, using a 2.5% H_2_/Ar ratio during growth. The growth parameters of samples A, B, C, and D are summarized in **Table** **1**, with additional details on the sample preparation process available in Supporting Information.

**Table 1:** Growth parameters of samples A, B, C, and D.

	Capping layer	Main layer	H_2_/Ar ratio during sputtering
Sample A	2 nm ZnO	40 nm Co(5 at%):ZnO	5%
Sample B	2 nm ZnO	40 nm Co(5 at%):ZnO	2.5%
Sample C	none	40 nm Co(5 at%):ZnO	5%
Sample D	none	40 nm ZnO	2.5%

## 2 Results and Discussion

**Figure 1:**
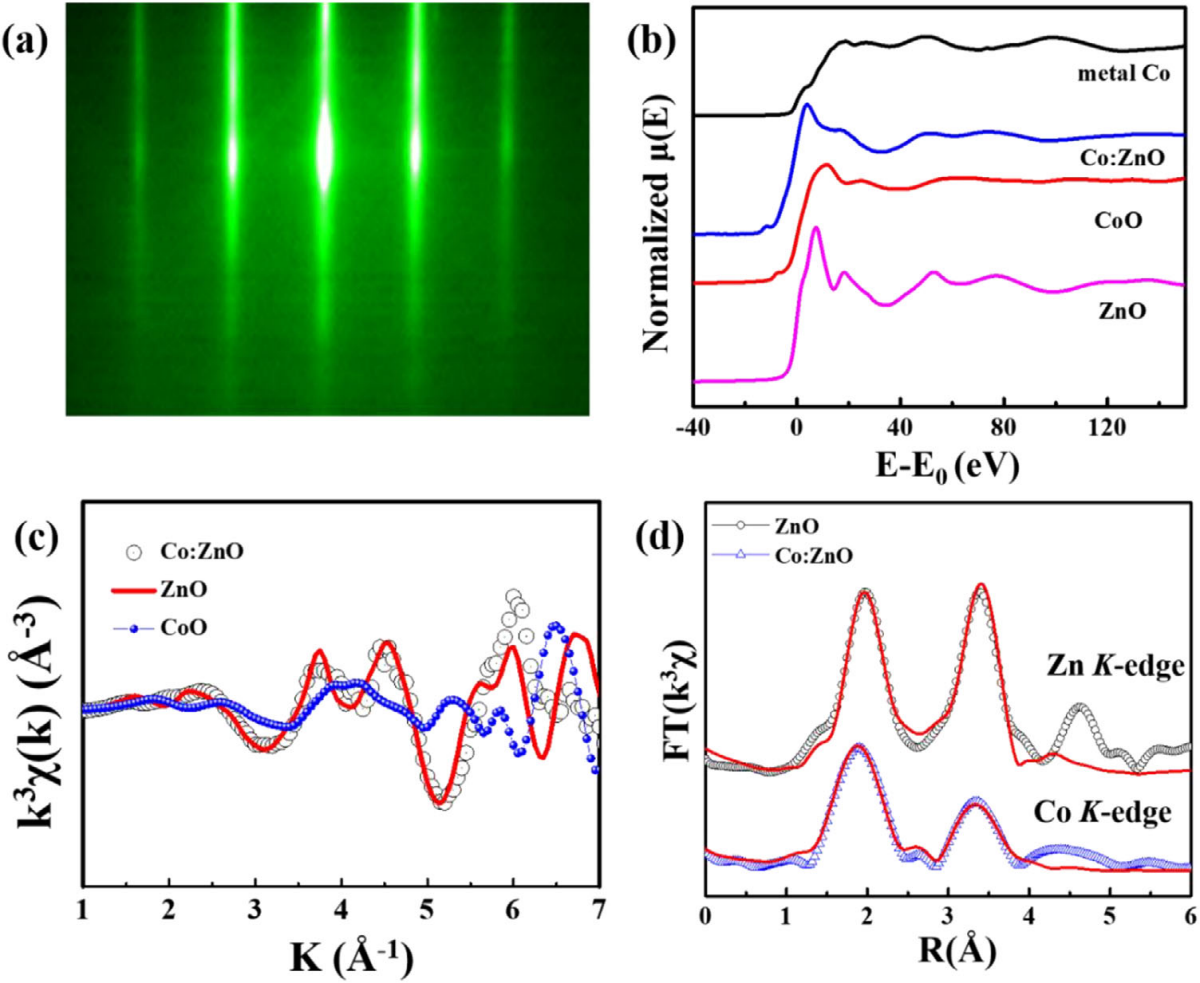
a) RHEED pattern of the Co_*n*_._*n*5_Zn_*n*_._95_O film along the [11‐20] azimuth, indicating high crystallinity. b) Normalized XANES spectra at the Zn *K*‐edge for undoped ZnO (sample D) and at the Co *K*‐edge for Co (5 at%) doped ZnO (sample A), compared with reference spectra from CoO powder and Co metal foil. c) *k^3^*‐weighted *χ*(k) functions at the Zn *K*‐edge (ZnO) and Co *K*‐edge (Co:ZnO), with CoO as reference. d) Fourier transformed EXAFS spectra of Co:ZnO at the Co *K*‐edge and ZnO at the Zn *K*‐edge. The overlaid fitting curve for Co:ZnO shows a local structural environment closely resembling that of Zn in ZnO.

**Figure** **1**a shows the reflection high‐energy electron diffraction (RHEED) pattern of Co_*n*_._*n*5_Zn_*n*_._95_O (sample A) along the [11‐20] azimuth, demonstrating high crystalline quality. Nearly identical RHEED patterns were observed for all samples, indicating similar structural properties throughout the series (see Figure S3, Supporting Information). To examine the local coordination and oxidation state of Co dopants, Co *K*‐edge X‐ray absorption near‐edge structure (XANES) spectra were collected at beamline BL12B2 of SPring‐8. As shown in Figure 1b, the Co *K*‐edge spectrum of Co:ZnO (sample A) exhibits a pre‐edge feature closely resembling that of CoO, indicating that Co atoms predominantly exist in the +2‐oxidation state. The Zn *K*‐edge spectrum of undoped ZnO (sample D) is shown for reference. This result supports the substitution of Co^2^⁺ at Zn^2^⁺ lattice sites, in agreement with previous studies.^[^ ^30^ ^]^ To further investigate the atomic structure, extended X‐ray absorption fine structure (EXAFS) measurements were performed at the Co *K*‐edge for Co:ZnO and the Zn *K*‐edge for ZnO. The background‐subtracted *µ*(E) data were converted to χ(k) using ATHENA from the IFEFFIT package.^[^ ^31^ ^]^ The *k^3^*‐weighted χ(k) functions were Fourier transformed using a Hann window over the k‐range of 1.0–7.0 Å^−^^1^, and the results are shown in Figure 1c. The corresponding radial distribution function (RDF) reveals that the Co‐O bond length in sample A is 1.93 ± 0.04 Å with a coordination number of 3.44 ± 1.6, which is comparable to the Zn‐O bond length of 1.95 ± 0.01 Å and coordination number of 3.57 ± 0.4 in sample D. In contrast, CoO^[^ ^32^ ^]^ exhibit Co‐O bond lengths of 2.12 Å and coordination numbers of 6. As illustrated in Figure 1d, the Fourier‐transformed EXAFS spectrum of Co:ZnO shows strong resemblance to that of ZnO, confirming that Co is incorporated substitutionally in the ZnO lattice rather than forming secondary phases. Similar structural features were observed in samples B and C (see Figure S4, Supporting Information), confirming consistent substitutional incorporation across all Co:ZnO films studied.^[^ ^26^ ^]^

**Figure 2:**
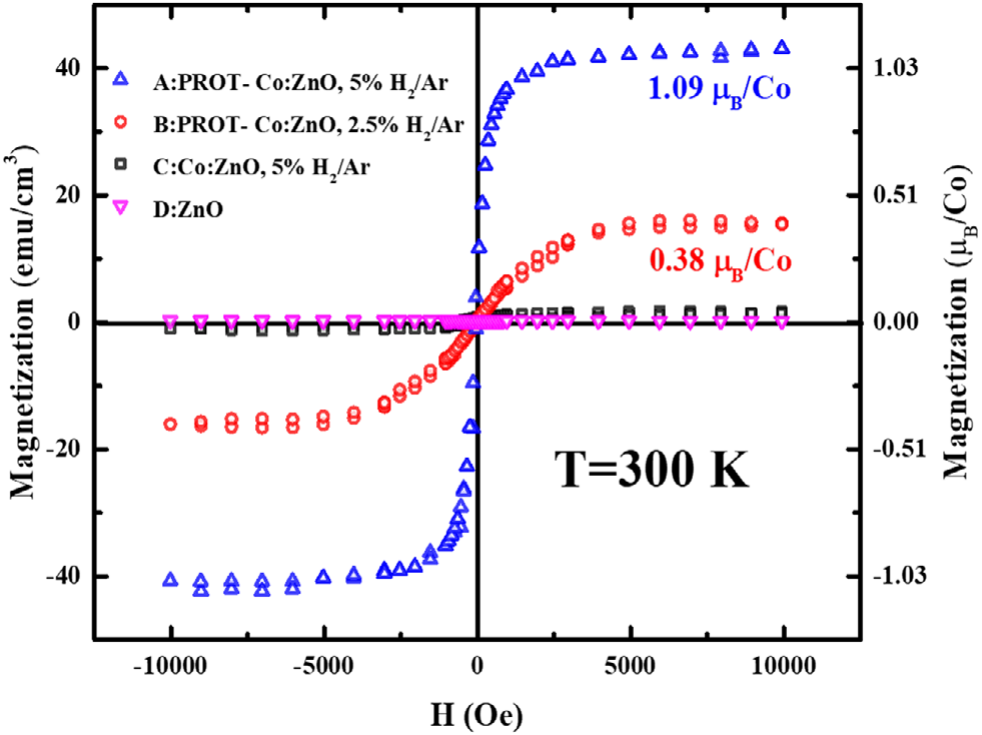
Magnetization of four samples at room temperature sample A, B, C, and D. The data have been corrected for the linear diamagnetic contribution of the sapphire substrates.

Room temperature magnetization (M) versus magnetic field (H) measurements along the in‐plane direction, using a superconducting quantum interface device (SQUID), confirm strong ferromagnetism in the Co:ZnO samples with a protection layer. After deposition, all samples were stored in a dry box for approximately three days prior to SQUID measurements. The M‐H hysteresis loops of sample A and B reveal saturation magnetization *Ms* of ≈1.09 and 0.38 *µ*_B_/Co, respectively, as shown in **Figure** **2**. On the other hand, the *Ms* values of sample C and sample D (ZnO) are less than 0.05 *µ*_B_/Co. These findings highlight the crucial role of both magnetic doping and structural defects in achieving RTFM in Co:ZnO. Furthermore, the magnetic properties of Co:ZnO without the protective layer (sample C) were monitored over time by exposing the samples to ambient conditions, as shown in Supporting Information. The results indicate a monotonic decay in ferromagnetism over time, reinforcing the critical role of both magnetic doping and structural defects in sustaining room‐temperature ferromagnetism (RTFM) in Co:ZnO. While similar findings have been reported in the literature, direct evidence for the presence of an impurity band in defect‐protected Co:ZnO remains absent.

**Figure 3:**
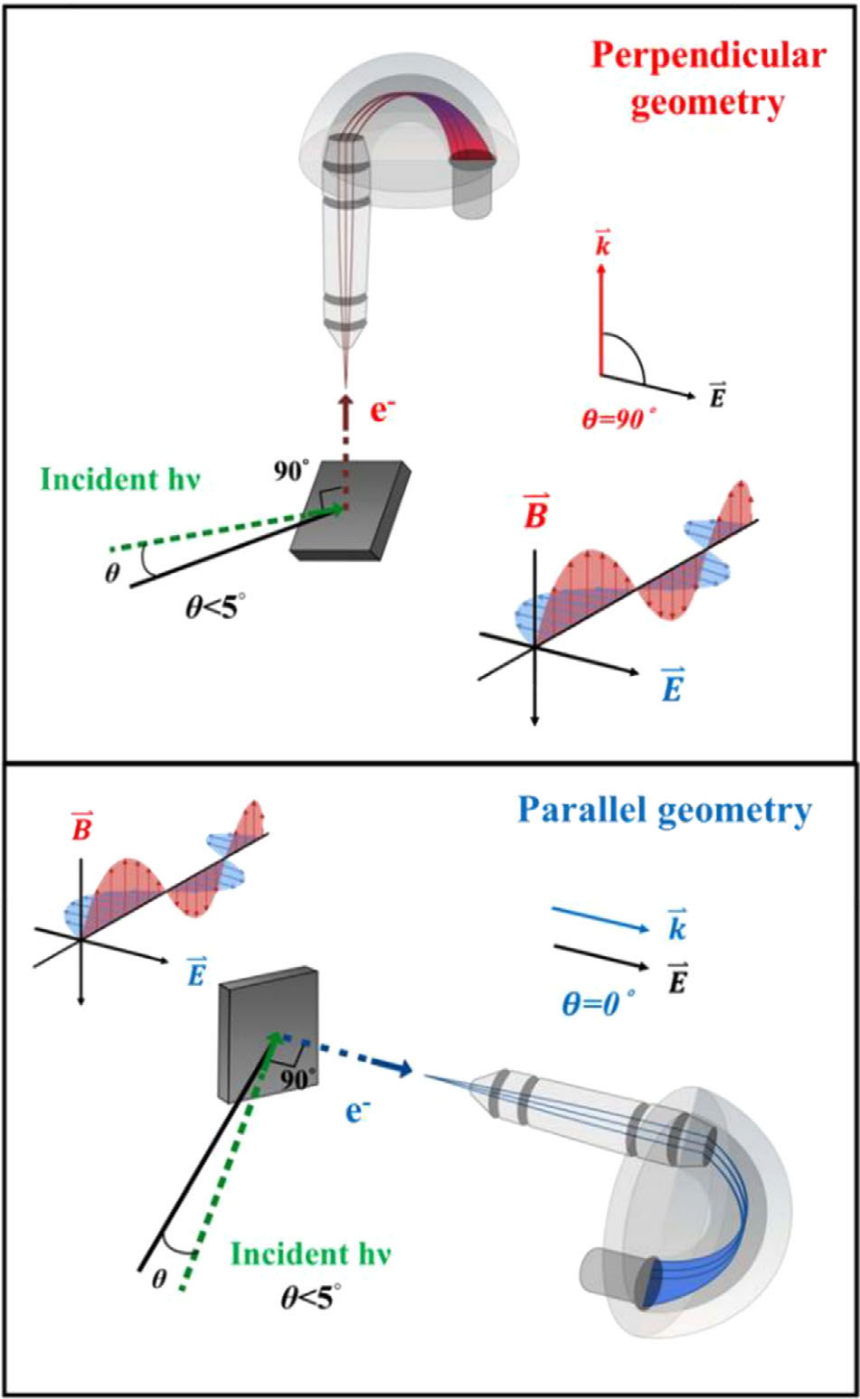
Experimental geometry of the polarization dependent HAXPES setup is defined by the angle *θ* between the incident photon beam electric field vector $\overset{⇀}{E}$ and the momentum $\overset{⇀}{k}$ of photoemitted electrons. The incident photon beam is linearly polarized with the electric field vector within the horizontal plane of the synchrotron ring. Top: In the perpendicular polarized geometry, (also called *s*‐polarized, where s stands for senkrecht≡perpendicular, in German) we use a vertically mounted analyzer, *θ =* 90°, and then $\overset{⇀}{k}$ is perpendicular to $\overset{⇀}{E}$. Bottom: In the parallel geometry, also called the *p*‐polarized geometry, we use a horizontally mounted analyzer, *θ =* 0°, and $\overset{⇀}{k}$ is parallel to $\overset{⇀}{E}$. Then, based on the angular dependence of differential ionization cross‐sections, one can distinguish features due to *s*, *p*, *d*,.‐type photoemitted electrons in a sample from the measured spectral intensities in the parallel and perpendicular geometries.

In the following we present direct evidence of the impurity band in defect‐protected Co:ZnO (Samples A and B) using HAXPES. HAXPES has proven to be a powerful tool for probing the bulk electronic structure of solids and thin films. A key advantage of using high photon energy is the enhanced probing depth, which enable access to intrinsic bulk electronic structure. Most importantly, polarization‐dependent HAXPES offers the capability to resolve the orbital character of electronic states in the valence band. In this study, polarization dependent HAXPES measurements were performed at the Max‐Planck‐NSRRC end‐station at the Taiwan undulator beamline BL12XU in SPring‐8, Japan. The experimental setup enables the independent detection of photoelectrons with momentum components both perpendicular and parallel to the electric field of nearly‐grazing incidence (<2°) synchrotron light, as schematically illustrated in **Figure** **3**.^[^ ^33^, ^34^ ^]^ The ability of polarization‐dependent HAXPES to distinguish electron states based on their orbital character makes it an invaluable technique for uncovering the electronic structure in DMSs and DMOs.

The angle‐dependent differential photoionization cross‐section is given by Trzhaskovskaya et al.^[^ ^35^, ^36^, ^37^, ^38^ ^]^

(1) $$\frac{d\sigma }{d\Omega }=\frac{\sigma }{4\pi }\left[1+\beta P_{2}\left(\cos\theta \right)+\left(\gamma \cos^{2}\theta +\delta \right)\sin\theta \cos\varphi \right]$$

where σ represents the photoionization cross section of a given subshell orbital, *P*_2_ is the second Legendre polynomial. The angle θ denotes the polar angle between the photoelectron momentum $\vec{k}$ and the polarization vector $\overset{⇀}{E}$ of the incident light, while φ signifies the azimuthal angle. This corresponds to a spherical coordinate system with the photon propagating along the x axis, θ as the polar angle and φ as the azimuthal angle. The parameters β, γ, and δ represent the angular distribution of the differential photoionization cross‐section. The first two terms in Equation (1) describe the dipole approximation, while the third term accounts for the non‐dipolar effects. Figure 3 depicts a schematic diagram of the experimental setup for the polarization dependent HAXPES measurements. In the perpendicular polarized geometry, using a vertically mounted analyzer, *θ =* 90°, and then the momentum $\overset{⇀}{k}$ of photoemitted electron is perpendicular to $\overset{⇀}{E}$. In contrast, in the parallel geometry, using a horizontally mounted analyzer, *θ =* 0°, and $\overset{⇀}{k}$ is parallel to $\overset{⇀}{E}$. Then, by analyzing the angular distribution of emitted photoelectrons, it becomes possible to determine their orbital character being *s*, *p*, *d*,…‐type electrons in a sample from the measured spectral intensities in the parallel and perpendicular geometries.

In particular, for the parallel geometry, since the analyzer axis is aligned along the electric field vector, θ = 0°, Equation (1) gets simplified to

(2) $$\frac{d\sigma }{d\Omega }=\frac{\sigma }{4\pi }\;\left\{1+\beta \right\}$$

and for the parallel geometry θ = 90°and φ = 90°, the Equation (1) is simplified to

(3) $$\frac{d\sigma }{d\Omega }=\frac{\sigma }{4\pi }\;\left\{1+\beta \left(\frac{1}{4}+\frac{3}{4}\cos2\theta \right)\right\}$$

**Figure 4:**
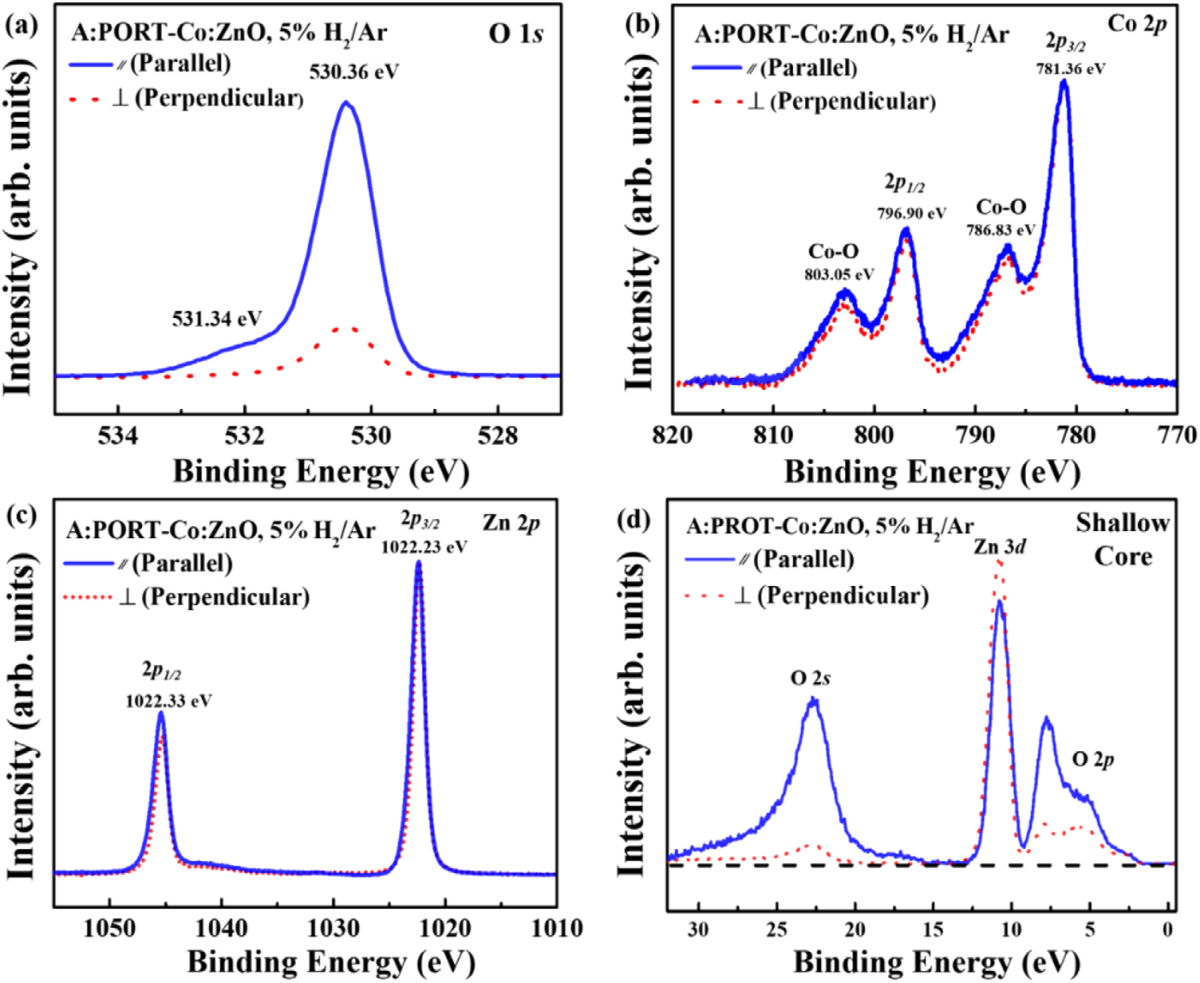
HAXPES spectrum of various core levels for sample A (PROT‐Co_0.05_Zn_0.95_O treated with 5% H_2_/Ar) measured at parallel and perpendicular geometry: a) O 1*s*, b) Zn 2*p*, c) Co 2*p*, and d) shallow core‐level region. All spectra are normalized to the intensity of the Zn 2*p* core level peak. Notably, when applying the same normalization factor of 0.25 as Zn 2*p* for Zn 3*d* states results in higher intensity for the Zn 3*d*_⊥_ spectral feature compared to Zn 3*d*_//_ spectral feature in Figure 4d.

Ideally, in the atomic limit, the angular distribution parameter β for *s* orbital is 2. Therefore, as shown in Figure S6, for the perpendicular geometry (θ = 90°), the emitted photoelectron intensity from *s* orbitals is expected to be zero. However, as shown by Weinen et al.^[^ ^33^ ^]^ for the same spectrometer, considering the acceptance angle of the analyzer slit, experimental β values for *s* orbitals are slightly less than 2, leading to a small but nonzero photoelectron intensity in the perpendicular geometry, which nonetheless remains significantly weaker compared to the parallel geometry. For NiO and ZnO, the authors reported that on normalizing at the Ni 2*p*/Zn 2*p* core levels, the O 1*s* spectra showed a parallel to perpendicular intensity ratio of 0.16.^[^ ^33^ ^]^ In **Figure** **4**a–d we show the polarization dependent HAXPES of core levels and the valence band of Co‐doped ZnO sample A. After normalizing at the Zn 2*p* peaks (Figure 4c), we confirmed that the O 1*s* spectra with the main peak at 530.36 eV is observed in both geometries in Figure 4a, while a weaker feature at 531.34 eV appears distinctly and is attributed to surface non‐stoichiometry.^[^ ^39^, ^40^ ^]^ On the other hand, since β values for *p* orbitals are typically close to 1,^[^ ^35^, ^36^, ^37^, ^38^ ^]^ from Equations [2] and [3], the actual experimental intensity ratio between the perpendicular and parallel geometry for the Zn 2*p* orbitals turns out to be ≈0.25. Figure 4b displays the HAXPES Co 2*p* core level spectra and notably, the spectra show no evidence of metallic Co. The main/satellite peak positions at 781.36 eV/786.83 eV and 796.9 eV/803.05 eV for the 2*p*_3/2_ and 2*p*_1/2_ indicate that Co^2+^ atoms are coordinated with oxygen, confirming Co substitution at the Zn site.^[^ ^39^, ^41^, ^42^, ^43^ ^]^ We have confirmed that the Co 2*p* HAXPES spectrum can be simulated by charge transfer multiplet configuration‐interaction (CI) model calculations using a tetrahedral [Co^2+^(O^2−^)_4_]^6−^ cluster and the results are detailed in Supporting Information, consistent with the XAS results discussed in Figure 1. Additionally, the calculations of the atomic orbital cross‐section (using interpolated angular distribution parameters from **Table** **2** ^[^ ^35^, ^36^, ^37^, ^38^ ^]^) yields a β value of ≈1.98 for Zn 4*s* at hυ=6.5keV. Thus, the expected photoelectron intensity ratio for Zn 4*s* orbitals between the perpendicular and parallel geometries is ≈0.0033, as derived from Equations (2) and (3). The shallow O 2*s* core level spectra, as well as the Zn 3*d*, O 2*p*, and Co 3*d* valence band features, are unambiguously identified through a systematic comparison between the spectra obtained under parallel and perpendicular linear polarization geometries,^[^ ^33^, ^43^, ^44^ ^]^ as shown in Figure 4d, and qualitatively consistent with tabulated values listed in Table 2. Specifically, the Zn 3*d* states manifest as a distinct peak at a binding energy of≈10 eV, consistent with the well‐localized semicore nature of the Zn 3*d* ^10^ configuration. The O 2*p* states dominate the valence band region between ≈3 and 8 eV and exhibit substantial hybridization with both Zn 4*s* and Co 3*d* states. Upon Co incorporation into the ZnO lattice, a prominent spectral shoulder emerges at the valence band maximum (VBM), between ≈1–3 eV, which is attributed to the Co^2+^ 3*d* ^7^ states. These Co 3*d* states, introduced via substitutional doping, hybridize with the neighboring O 2*p* orbitals and form a partially occupied impurity band near the conduction edge, as also reported in prior investigations of Co‐doped ZnO systems.^[^ ^45^ ^]^

**Table 2:** Subshell photoionization cross section (σ) in kb (= 10^−21^ cm^2^) at 6.5 keV, deduced from refs.[35, 36, 37, 38] The cross sections for parallel and perpendicular geometries are calculated using Equations [2] and [3] with θ = 0° and θ = 90°, respectively.

Atomic subshell	σ/e^−^ [10^−3^ kb]	β	Para. [10^−3^ kb]	Perp. [10^−3^ kb]
Zn4*s*_1/2_ (E_b_ = 9 eV)	26.25	1.98	78.22	0.26
Zn3*d*_3/2_ (E_b_ = 12 eV)	3.15	0.45	4.57	2.44
Zn3*d*_5/2_ (E_b_ = 11 eV)	4.51	0.46	6.58	3.47
Co3*d*_3/2_ (E_b_ = 9 eV)	1.53	0.34	2.06	1.27
Co3*d*_5/2_ (E_b_ = 9 eV)	1.47	0.36	2.00	1.21
O2*p*_1/2_ (E_b_ = 14 eV)	0.20	0.09	0.22	0.19
O2*p*_3/2_ (E_b_ = 14 eV)	0.20	0.10	0.22	0.19
O2*s*_1/2_ (E_b_ = 28 eV)	22.88	1.96	67.72	0.46

**Figure 5:**
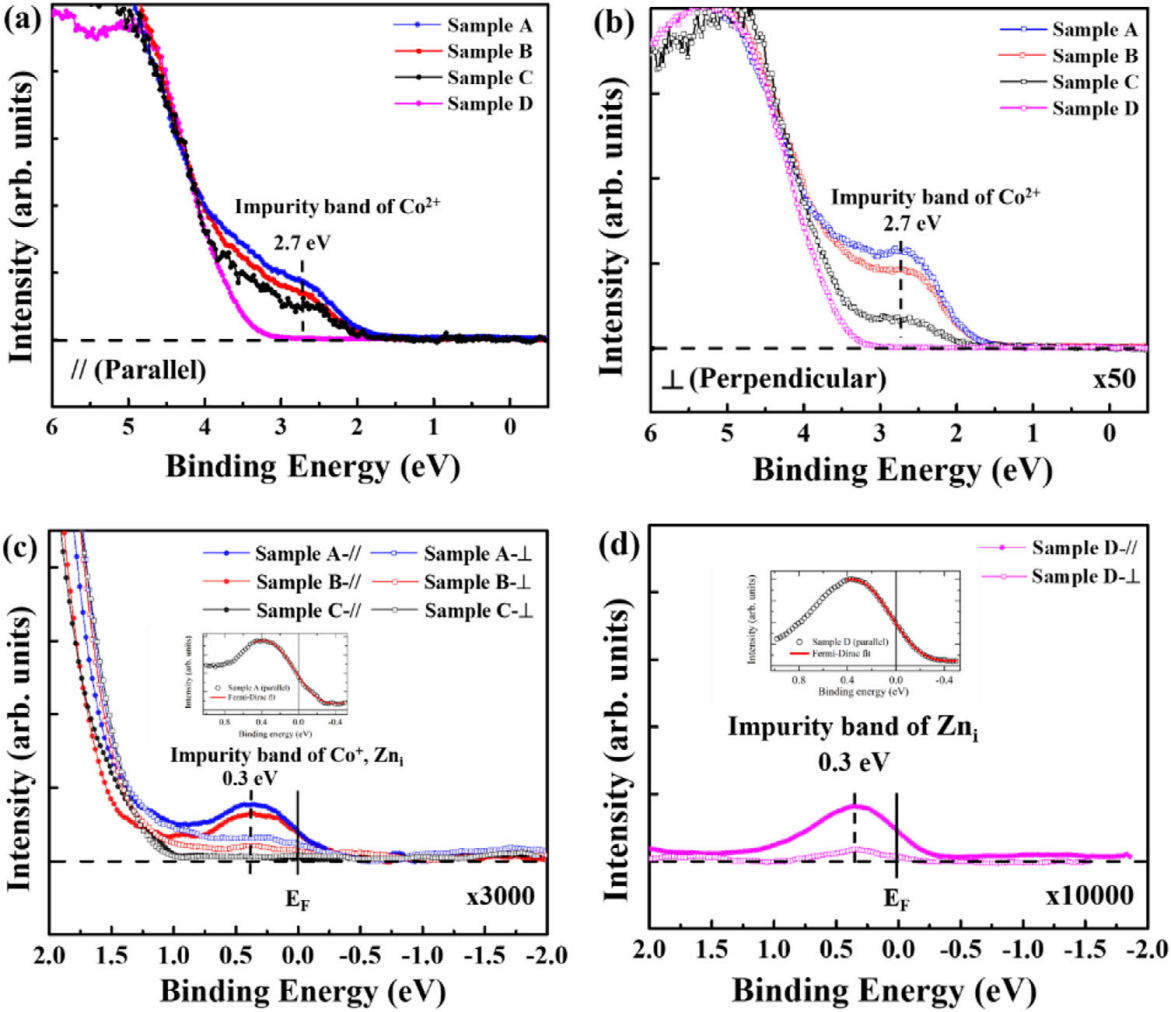
a) Valence band HAXPES spectrum measured under parallel geometry, serving as the reference for the y‐axis scale across all panels in this figure. b) Magnified view (×50) relative to the y‐axis scale of (a), showing the valence band HAXPES spectrum acquired under perpendicular geometry. c) Highly magnified view (×3000) relative to the y‐axis scale of (a), comparing the valence band HAXPES spectra of samples A, B, and C measured under both parallel and perpendicular geometries. Inset: Fermi edge observed in sample A under parallel geometry. d) Further magnified view (×10 000) relative to the y‐axis scale of (a), displaying the valence band HAXPES spectra of sample D under both parallel and perpendicular geometries. Inset: Fermi edge observed in sample D under parallel geometry.

In ZnO‐based DMOs, structural defects primarily consist of zinc interstitials and oxygen vacancies.^[^ ^46^, ^47^, ^48^, ^49^, ^50^ ^]^ As a result, the impurity bands associated with these defects and Co‐doping in Co:ZnO samples are expected to originate either from Zn 3*d*/4*s*, Co 3*d* and O 2*p* orbitals or from a possible hybridization/interplay of these electronic states. Based on Table II, it is expected that the spectral weight associated with these states will be enhanced in the parallel geometry of polarization‐dependent HAXPES measurements. In the following, we systematically investigate the valence band states at and in the vicinity of *E_F_* to identify the character of observed spectral features in Samples A to D. **Figure** **5**a,b present the HAXPES data for the parallel and perpendicular geometries within 6 eV BE of samples A through D, revealing a band at ≈2.7 eV but with significantly different spectral intensities. Since the intensity of this feature is weaker in the perpendicular geometry, but follows the same trend in parallel and perpendicular geometries, i.e., it is maximum for sample A, and then gets reduced for Sample B and gets further reduced for sample C, while the feature is totally missing in sample D, it follows the magnetization magnitude of the samples and indicates that the 2.7 eV BE feature is due to Co 3*d* states. It is also noted that although sample B exhibited a lower defect concentration (H_2_/Ar ratio of 2.5%), it was protected by a capping layer, whereas sample C, with a higher defect concentration (H_2_/Ar ratio of 5%), remained uncapped. These findings highlight the critical role of the capping layer in preserving the ferromagnetic properties, indicating that protection from environmental factors is essential, even when intrinsic defect densities are relatively low Since the long range ordered high‐*T*c samples A and B are nearly metallic, we carried out a very high signal to noise ratio examination of the HAXPES data at and near *E_F_* to check for the donor band, as shown in Figure 5c,d. We first discuss the results for samples A‐C shown in Figure 5c. The data indeed reveals a band peaked at ≈0.3 eV below *E_F_* in samples A and B and crosses *E_F_* with finite intensity, but this feature is missing in sample C. More importantly, the 0.3 eV peak gets strongly suppressed in perpendicular geometry for samples A and B. It is important to note that the HAXPES signal associated with Zn 4*s* orbitals is reduced by the polarization factor in perpendicular geometry. Thus, from the magnitude of its spectral weight reduction, and the fact that the feature is missing in sample C which includes Co 3*d* states at 2.7 eV BE as seen in Figure 5a,b, it indicates that the 0.3 eV feature originates in a dominantly Zn 4*s* character metallic donor band as measured by HAXPES. The O 2*p* character is also ruled out as Table 2 indicates nearly similar spectral intensity is expected for O 2*p* states in parallel and perpendicular geometry.

Finally, to validate the Zn 4*s* character of the donor band, we measured polarization dependence of sample D as shown in Figure 5d. The results reveal an electron donor band even for sample D and while it is weaker than the donor band seen in samples A and B, it shows a qualitatively similar polarization dependence with strongly suppressed spectral intensity in perpendicular geometry, just like the electron donor band seen in Co‐doped ferromagnetic samples A and B. Since sample D does not contain Co 3*d* dopants, this result unambiguously indicates that the donor band originates in Zn 4*s* character electrons.

In bulk ZnO, the ionic charge‐transfer model describes how Zn 4*s* electrons populate unfilled O 2*p* states, leading to the formation of the Zn^2^⁺O^2^^−^ chemical state. The starting chemical configuration of Zn is 3*d* ^1^⁰4*s* ^2^, while that of O is 2*s* ^2^2*p*⁴. Due to simple charge transfer and formation of Zn^2+^ and O^2−^ states, Zn 4*s* orbitals hand over electrons to fill the the O 2*p* band, preserving the characteristic valence band structure with a strongly stabilized fully filled Zn 3*d* band at ≈11 eV BE. However, in ZnO films containing structural defects, such as Zn interstitials or O vacancies, Zn 4*s* electrons become available to form an “*s*”‐electron character donor band near or at the Fermi level, leading to metallic behavior.^[^ ^48^, ^49^, ^50^ ^]^ In ZnO (sample D), as shown in Figure 5d, a faint HAXPES spectral feature near the Fermi energy cutoff originates from the filling of Zn 4*s* states playing a dominant role. Similarly, when Co is introduced into ZnO with sufficient structural defects, it forms a distinct electron donor band but with higher spectral weight, as shown in Figure 5c. To further explore the orbital nature of this donor band, we performed density functional theory (DFT) calculations on a ZnO supercell incorporating a substitutional Co atom and two neighboring oxygen vacancies. The resulting Co 3*d* and Zn 4*s* partial density of states (PDOS) confirm the formation of a hybridized donor band at *E_F_* as discussed in Supporting Information and presented in Figure S8. The theoretical results reveal that oxygen vacancies promote *s‐d* hybridization between Zn 4*s* and Co 3*d* orbitals, leading to the formation of a spin‐polarized impurity band that facilitates long‐range magnetic coupling. These findings are in excellent agreement with our spectroscopic and magnetic measurements. Two scenarios have been postulated to explain the origin of the electron donor band in Co‐ doped ZnO: In one case, the donor band is capable of binding electrons, leading to localized bound magnetic polaron (BMP)^[^ ^11^, ^51^ ^]^ states around each Co ion. In the second case, the donor band can form a delocalized metallic band which can facilitate Ruderman‐Kittel‐Kasuya‐Yosida (RKKY)‐type long range exchange interactions between dopant Co^2+^ ions. The present polarization dependent HAXPES measurements provide direct evidence of an impurity donor band in Co‐doped ZnO, in the form of Zn 4*s* character metallic band crossing the Fermi level and favors the RKKY picture for the high‐T_C_ ferromagnetism in Co‐doped ZnO. The present finding thus offers a new insight into the ferromagnetic mechanism, where Zn^1+^4*s* ^1^ states mediate the ferromagnetism in Co‐doped ZnO.

## 3 Conclusion

Our study resolves a fundamental paradox in dilute magnetic oxides, where the use of a protective layer is essential for preserving structural defects but simultaneously affects surface‐sensitive signal measurements. Unlike conventional surface‐sensitive techniques such as XMCD and EMCD, polarized HAXPES enables the detection of both bulk and orbital characteristics. We demonstrate the application of a protective layer effectively preserve structural defects, allowing for a more accurate investigation of their role in the electronic and magnetic properties in DMOs.

By overcoming limitations of measurement techniques and sample preparation challenges, we successfully observe the impurity band arising from the exchange interaction between transition metal elements and structural defects. This finding provides crucial insights into the mechanism of RTFM in DMOs. The incorporation of 3*d* transition metals into functional semiconductor materials to induce magnetism has long been a topic of considerable interest due to its promising technological applications. A key aspect of this lies in understanding how charge carriers in the host semiconductor interact with the *d*‐electrons of dopant atoms. Our research not only sheds light on the mechanism behind the magnetic properties in the Co:ZnO system but also offers significant contributions to the methodologies used for studying the incorporation of 3*d* transition metals into semiconductors.

### 3.1 Sample Preparation

Co:ZnO thin films were grown by radio frequency magnetron sputtering (RF sputter) which provide large size uniform samples on sapphire substrates. The sapphire substrates were cleaned with a standard procedure before loading into the growth chamber, the sapphire substrate was heated to 1000 °C for 1 h. The base pressure of the RF‐Sputtering system (AdNaNo Corp.) was less than 2 × 10^−10^ torr; the growth pressure of the Co:ZnO thin film was maintained below 5 × 10^−3^ torr. The growth power was controlled at 75 watts. The substrate was maintained at 300 °C during the growth. Oxygen vacancies were created during growth by using Ar mixed H_2_ atmosphere. We fabricated the Ar 95% mixed H_2_ 5% and Ar 97.5% mixed H_2_ 2.5% to compare the trend of oxygen vacancy. For prevent oxygen vacancy was filled in air surrounding again that grown the Co_0.05_Zn_0.95_O in Ar working pressure.

### 3.2 X‐Ray Absorption Spectroscopy Characterization

The local structure of Co doped ZnO thin films was determined using XANES and EXAFS spectra. The *K*‐edge spectra of Co (7709 eV) and Zn (9659 eV) were recorded at room temperature at Taiwan beamline SP12B2 of SPring‐8. The fluorescence mode was implemented with the beam incident at 54.7 degrees with respect to the sample plane; the signal was collected with a Lytle detector. The measured energy resolution of the double‐crystal Si(111) monochromator was better than 0.6 eV.

### 3.3 Magnetization Characterization

The M‐H magnetization curves were measured in a Quantum Design magnetic properties measurement system (MPMS). The superconducting quantum interference device magnetometer provides ≤10^−8^ emu sensitivity and a magnetic field of up to 7 Tesla.

### 3.4 Hard X‐Ray Photoemission Spectroscopy Characterization

The polarization dependent HAXPES experiments were carried out at the Max‐Planck‐NSRRC end station at the Taiwan undulator beamline BL12XU at SPring‐8, Japan. The photon energy was set to 6.5 keV. The experimental setup has two MB Scientific A‐1 HE analyzers, for parallel and perpendicular geometry measurements. The overall energy resolution was 0.27 eV as determined from the Gaussian broadening used to simulate the Fermi edge of gold. All measurements reported here were performed at room temperature.

## Conflict of Interest

The authors declare no conflict of interest.

## Author Contributions

P.‐Y.C., Y.‐F.L., and J.‐C.‐A.H. conceived and planned the experiments. P.‐Y.C., Y.‐F.L., and C.‐Y.S. carried out the experiments. A.A.C. planned and carried out the simulations. C.‐H.L. provided the DFT band structure calculations. C.‐Y.S., C.‐H.L., and C.‐Y.L. contributed to sample preparation. P.‐Y.C., Y.‐F.L., K.‐D.T., A.A.C., H.‐S.H., and J.‐C.‐A.H. contributed to the interpretation of the results. P.‐Y.C., H.‐S.H., A.A.C., and J.‐C.‐A.H. took the lead in writing the manuscript. All authors provided critical feedback and helped shape the research, analysis, and manuscript

## Supporting information



Supporting Information

## Data Availability

The data that support the findings of this study are available from the corresponding author upon reasonable request.
