# Supplementary material for: An “s‐Electron” Donor Band Driven Metallic Ferromagnetism in Co‐Doped ZnO Films
Source: Adv Sci (Weinh). 2025 Jul 26;12(40):e08148. doi: 10.1002/advs.202508148 (PMC12561263; doi:10.1002/advs.202508148)
Supplement: Supplementary file 1 — Supporting Information [file ADVS-12-e08148-s001.docx]

Supporting Information

**An “*s*-electron” donor band driven metallic ferromagnetism in Co-doped ZnO films**

Pei-Yu Chuang^*^, Jung-Chun-Andrew Huang^*^, Ashish Atma Chainani^*^, Hua-Shu Hsu, Yen-Fa Liao, Chang-Yi Sung, Chih-Hua Liu, Chien-Yu Liao, Chi-Hsuan Lee, and Ku-Ding Tsuei

P.-Y. Chuang, J. C. A. Huang, C.-Y. Sung, C.-H. Liu, C.-Y. Liao

Department of Physics, National Cheng Kung University, Tainan 701, Taiwan

E-mail: [jcahuang@mail.ncku.edu.tw](mailto:jcahuang@mail.ncku.edu.tw)

P.-Y. Chuang, A. A. Chainani, Y.-F. Liao, K.-D. Tsuei

National Synchrotron Radiation Research Center, Hsinchu 300, Taiwan

E-mail: [chuang.py@nsrrc.org.tw](mailto:chuang.py@nsrrc.org.tw); [chainani.ash@nsrrc.org.tw](mailto:chainani.ash@nsrrc.org.tw)

J. C. A. Huang

Department of Applied Physics, National University of Kaohsiung, Kaohsiung 81100, Taiwan

J. C. A. Huang

Taiwan Consortium of Crystalline Materials (TCECM), National Science and Technology Council, Taipei 106, Taiwan

H.-S. Hsu

Department of Applied Physics, National Ping Tung University, Pingtung 90003, Taiwan

C.-H. Lee

Research Center for Applied Sciences, Academia Sinica, Taipei 11529, Taiwan

**Supplementary Ι: The ZnO encapsulated Co:ZnO films are referred to as protected Co:ZnO (PROT-Co:ZnO) sample**s

Following hydrogen treatment, Co:ZnO and ZnO samples with high defect concentrations were employed to monitor the decay of defect density over time under ambient conditions. To provide protection, ZnO films with a low defect concentration were deposited atop the samples, as depicted in Fig. S1


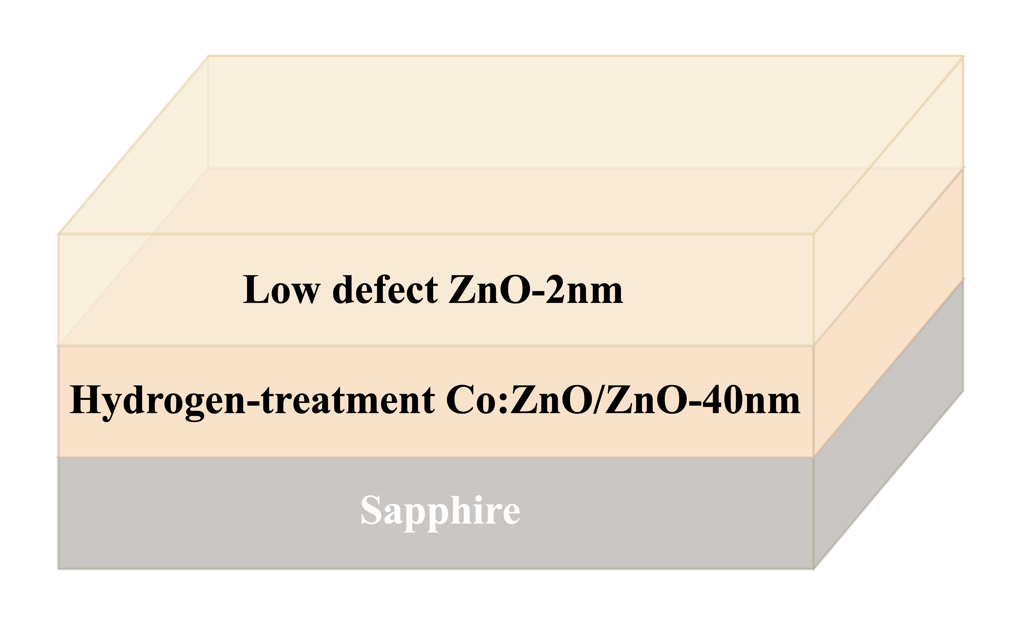


**Figure S1.** A 2nm-thick ZnO protective layer with a low defect concentration encapsulated the 40nm-thick Co:ZnO and ZnO films with high defect concentrations.

To ensure the HAXPES measurement, the specimens must be sufficiently conductive. Since ZnO thin films possess a wide band gap of approximately 3.4 eV, rendering single crystals insulating, precautions are necessary to mitigate surface charging effects during HAXPES analysis. To address this, Sample D of ZnO was hydrogenated using a 2.5% H_2_/Ar ratio during sputtering, resulting in the formation of the Zn 4*s* band near the Fermi level. To further illustrate this effect, ZnO subjected to excessive hydrogenation with a 7.5% H_2_/Ar ratio exhibit a significantly broadened defect band, as illustrated in Figure S2. However, in this case, the Wurtzite structure of ZnO was disrupted due to an excesses number of defects.


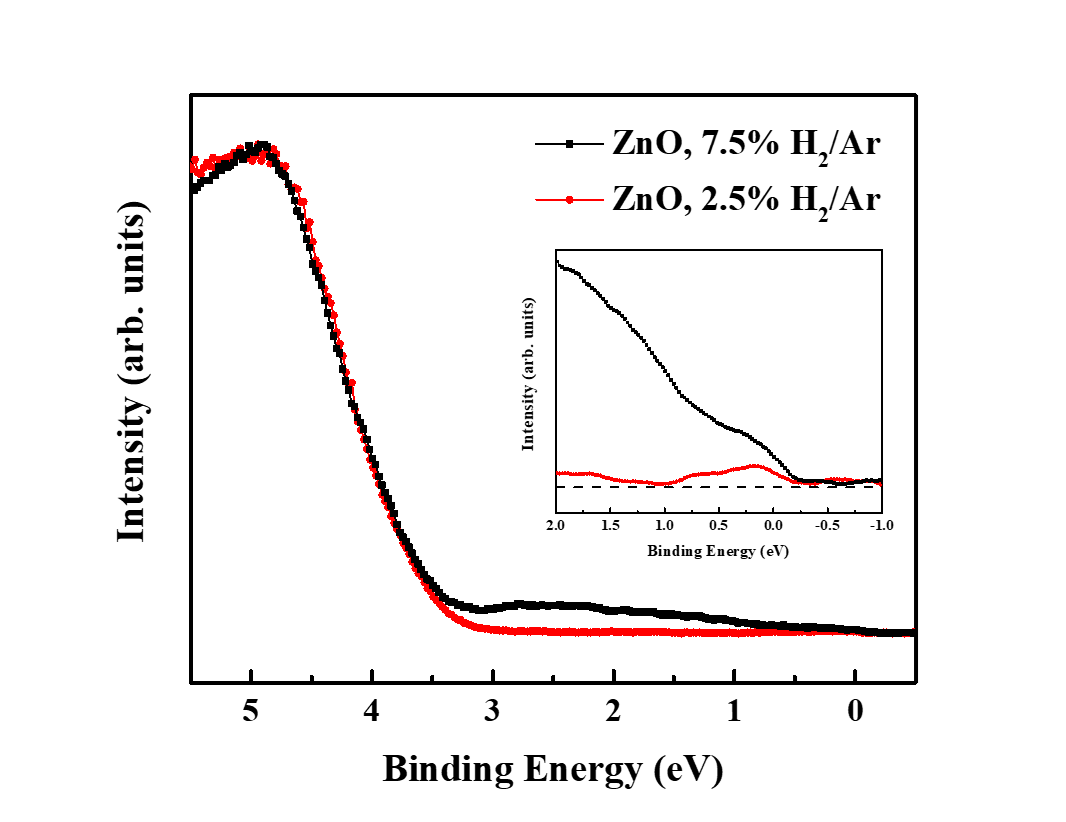


**Figure S2.** HAXPES spectra of hydrogenated ZnO samples treated with H₂/Ar gas mixtures at 2.5% and 7.5%, respectively. The spectra were measured under *p*-polarized geometry (parallel geometry), with the 2.5% sample denoted by a red circle and the 7.5% sample by a black square. **Inset:** Magnified view of the spectral region between -2 eV and -1 eV, highlighting the relative intensity enhancement and peak evolution associated with defect-related states and hydrogen-induced shallow donor levels.

**Supplementary Ⅱ:** **Structural Characterization of Co:ZnO**

To assess the crystalline quality of all ZnO-based thin films studied, we present in Fig. S3 the reflection high-energy electron diffraction (RHEED) patterns of samples A, B, and C (Co:ZnO films grown under various H₂/Ar ratios) and the ZnO reference (sample D). All images were obtained along the [11-20] azimuth. The presence of well-defined streaks in each pattern confirms a smooth surface and two-dimensional growth mode. Furthermore, the similarity in diffraction features among the Co-doped and undoped samples suggests that cobalt doping and varying hydrogen partial pressure do not introduce significant crystalline disorder. These results affirm that epitaxial growth is preserved across all conditions, supporting the structural integrity of the films used for subsequent spectroscopic and magnetic analyses. To verify that Co atoms in samples B and C exhibit similar local structural environments to those in sample A, we performed Co *K*-edge EXAFS measurements on all three samples. The *k*³-weighted Fourier-transformed spectra are shown in Fig. S4, along with reference spectra for CoO and metallic Co. All three Co:ZnO samples exhibit a primary coordination peak centered at ~1.9 Å, corresponding to the Co-O bond distance in the wurtzite ZnO lattice. This feature is clearly distinct from the Co-O (~2.1 Å) peak observed in CoO. The consistent spectral profiles across samples A, B, and C confirm that Co atoms are substitutionally incorporated at Zn sites in all Co-doped ZnO films.


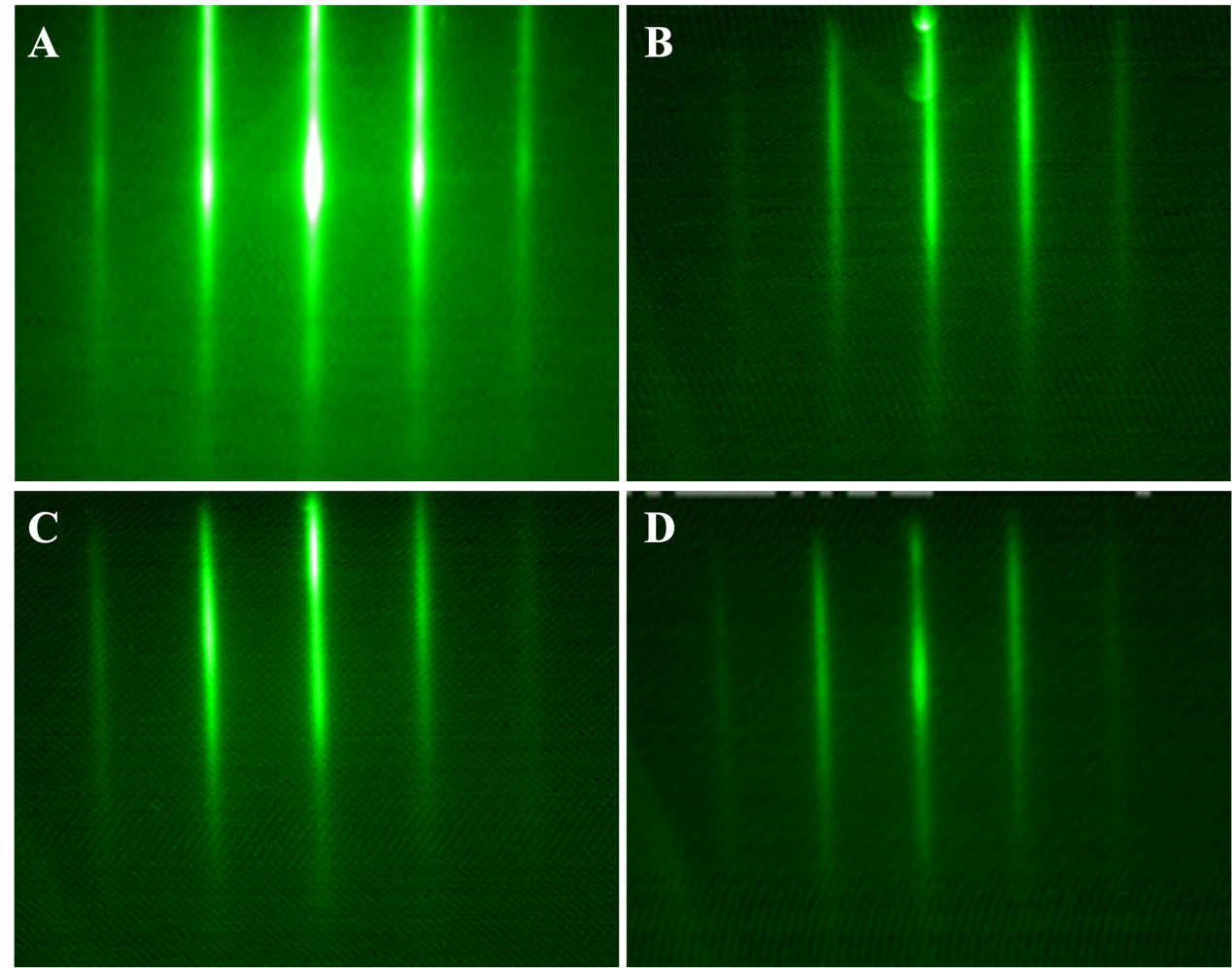


**Figure S3**. RHEED patterns of (A) PROT- Co₀.₀₅Zn₀.₉₅O, 5% H_2_/Ar (sample A), (B) PROT- Co₀.₀₅Zn₀.₉₅O, 2.5% H_2_/Ar (sample B), (C) Co₀.₀₅Zn₀.₉₅O, 5% H_2_/Ar (sample C), and (D) ZnO (sample D), acquired along the [11-20] azimuth. All samples exhibit streaky diffraction features indicative of smooth film surfaces and epitaxial growth. The similarity of the patterns across Co-doped and undoped films confirms that hydrogen-assisted growth conditions do not degrade the overall crystalline quality of the ZnO-based thin films.


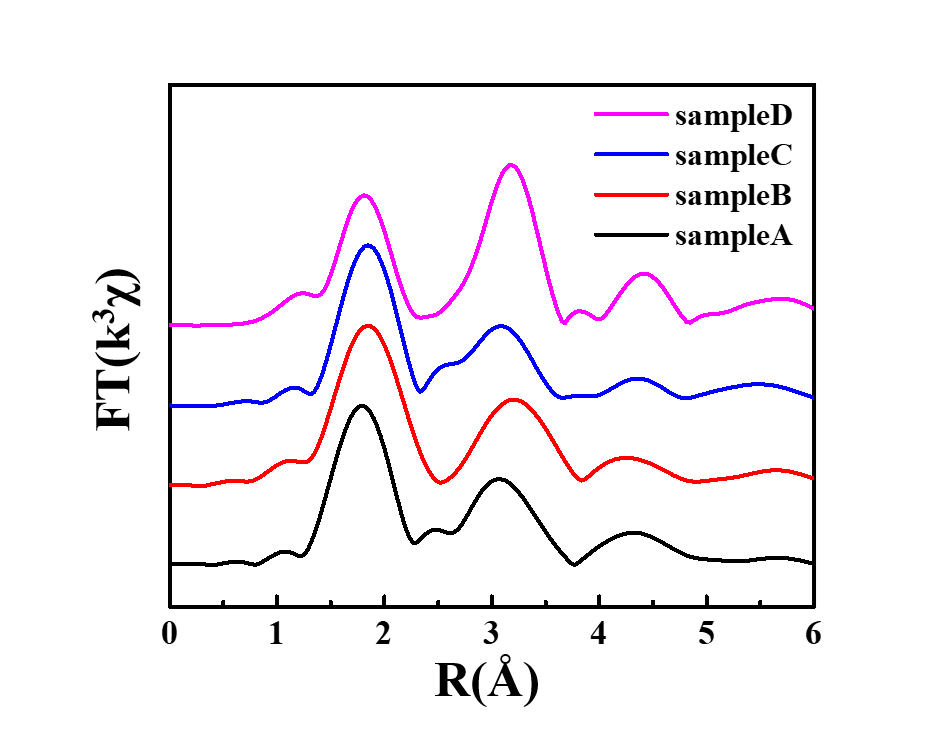


**Figure S4.** Fourier-transformed EXAFS spectra at the Co *K*-edge for Co:ZnO samples A, B, and C, along with reference spectra of ZnO (sampleD). All spectra were obtained after *k*³-weighted transformation over the range of 1.0-7.0 Å^-^¹ using a Hann window. The main coordination peak observed at ~1.9 Å for samples A, B, C corresponds to Co-O bonding in the wurtzite ZnO lattice, and is clearly distinguishable from the Co-O bond distances in ZnO. The similarity among samples A, B, C confirms the substitutional incorporation of Co at Zn sites across all Co:ZnO films.

.

**Supplementary** **Ⅲ: The magnetic properties of Co:ZnO without the protective layer by exposing the samples to ambient conditions**.

Previous studies have indicated that structural defects, such as oxygen vacancies or Zn interstials, tend to destabilize under ambient conditions. To study this effect, we compared the ferromagnetism of Co_0.05_Zn_0.95_O sample (without protective layer)- fabricated using RF sputtering treated with 5% H_2_/Ar and annealed for 8 hours at 250^o^C- immediately after growth and after 5-days of air exposure, as illustrated in Fig. S5. The observed reduction in magnetization is likely due to the refilling of structural defects by atmospheric oxygen. To prevent the degradation of magnetic properties under ambient conditions, a ZnO protective layer with a low defect density was deposited on top of the samples. This protective layer poved essential in preserving the room temperature ferromagnetism.





**Figure S5.** The magnetization of the 40 nm thick (Co_0.05_Zn_0.95_)O film immediately after growth (black square) and after 5-days of air exposure (red circle). The data have been adjusted to account for the linear diamagnetic contribution of the substrate.



**Figure S6.** For linearly polarized light, angular intensity distribution of photoemitted electrons depends on the asymmetry parameter β>0 at energies of several keV, for almost all subshells J. Yeh & I. Lindau At. Data. Nucl Data Tables 32, 1(1985) Their intensities have a maximum in a direction parallel to the electric polarization vector.

**Supplementary** **Ⅳ: The configuration-interaction (CI) cluster-model calculation of Co 2*p* core level spectrum.**

We have carried out charge transfer multiplet configuration-interaction (CI) cluster-model analysis of the Co-2*p* spectrum using a tetrahedral [Co^2+^(O^2-^)_4_]^6-^ cluster. The initial and final states are described by a linear combination of the *d*^7^, *d*^8^L^1^, and *d*^9^L^2^ states, following the methodology detailed in ^[1]^. The calculated spectrum showed a reasonable agreement with the experimental spectrum, as shown in Fig. S7, using the following electronic structure parameters: ligand-to-3*d* charge transfer energy Δ= 6.0 +1eV, on-site *d-d* Coulomb interaction energy *U_dd_* = 6.0 eV, core hole potential *U_pd_* = 8.5 eV, hybridization strengths V_eg_ = 2.9 eV, V_t2g_ = 1.5 eV and crystal field splitting 10*D*q = 0.7 eV. These parameters are closely aligned with those reported in a similar calculation of the Co 2*p* spectrum obtained by soft x-ray PES ^[2]^ and are consistent with the established chemical trends in transition-metal-doped II-VI diluted magnetic semiconductors ^[3]^.


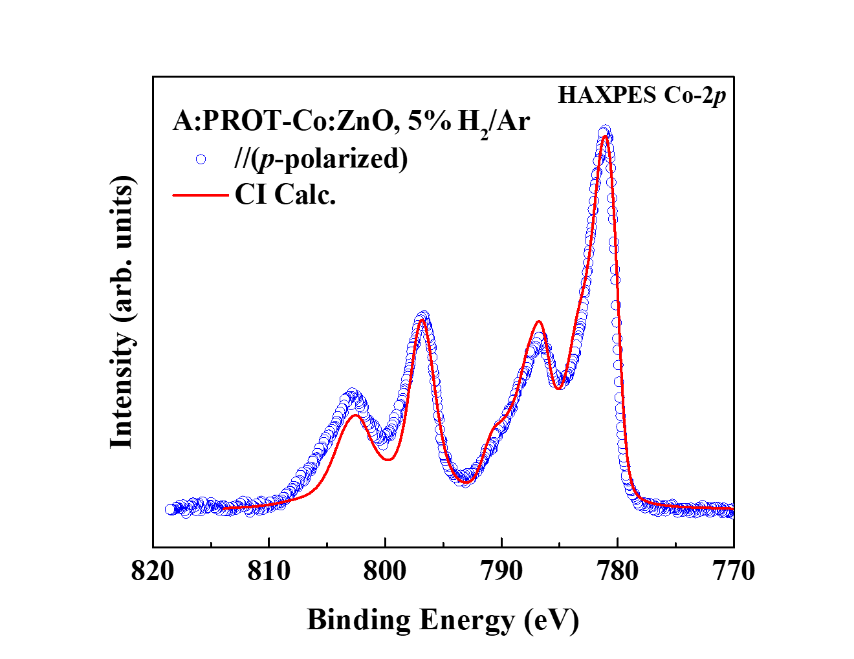


**Figure S7.** The Co-2*p* HAXPES spectrum of sample A (PROT-Co:ZnO, 5% H_2_/Ar) is shows as blue circle dots, compared with the CI cluster-model calculation represented by the red line. The comparison demonstrates a reasonable agreement between the experimental and calculated spectra.

**Supplementary V: First-Principles Calculations on Oxygen Vacancy Induced Ferromagnetism in Co-Doped ZnO**

To complement the experimental investigation and provide deeper insight into the microscopic origin of ferromagnetism in Co-doped ZnO, first-principles density functional theory (DFT) calculations were performed using the Vienna Ab initio Simulation Package (VASP).^[4]^ The electronic structure was calculated within the generalized gradient approximation (GGA) framework, employing the Perdew–Burke–Ernzerhof (PBE) exchange-correlation functional.^[5]^ A 48-atom wurtzite ZnO supercell was used, with one Zn atom replaced by Co to simulate a 5 at% doping concentration. Oxygen vacancies were introduced via special quasi-random structures (SQS) generated using a Monte Carlo approach.^[6]^

The spin-polarized density of states (DOS) results show that Co substitution alone yields localized magnetic moments; however, significant ferromagnetic coupling only emerges in the presence of oxygen vacancies. These vacancies induce local lattice distortion and enhance the hybridization between Co 3*d* and Zn 4*s* orbitals. The Zn 4*s* states shift toward the Fermi level under increasing vacancy concentration and become weakly spin-polarized. This *s-d* hybridization is consistent with the donor-mediated exchange mechanism proposed by Walsh et al..^[7]^ As shown in Fig. S8, systems with higher oxygen vacancy content exhibit an upward shift of the Fermi level, leading to partial population of the conduction band. This facilitates the formation of spin-polarized Zn 4*s*-Co 3*d* hybrid states, which can support long-range magnetic interactions through an extended impurity band. These theoretical results align well with the experimental observations from HAXPES and SQUID measurements, reinforcing the conclusion that defect engineering particularly the stabilization of donor-like states is essential for realizing robust room-temperature ferromagnetism in Co-doped ZnO.

Furthermore, the projected density of states (PDOS) analysis indicates that the Zn 4*s* PDOS at the Fermi level (*E_F_*) is approximately 0.32 states/eV, while the Co 3*d* PDOS at *E_F_* is about 11.728 states/eV. Although the Co 3*d* PDOS is 36.65 times greater than that of Zn 4*s*, the photoionization cross-section (PICS) observed in HAXPES reveals that the Zn 4*s* contribution dominates, with a Zn 4*s*:Co 3*d* intensity ratio of roughly 38.53. This apparent inconsistency underscores the significant role of photoemission matrix elements and highlights that Zn 4*s* states, albeit low in PDOS, contribute strongly to the spectral weight near *E_F_* in HAXPES measurements. Therefore, the observed spectral intensity near *E_F_* is predominantly attributed to Zn 4*s* derived donor states.


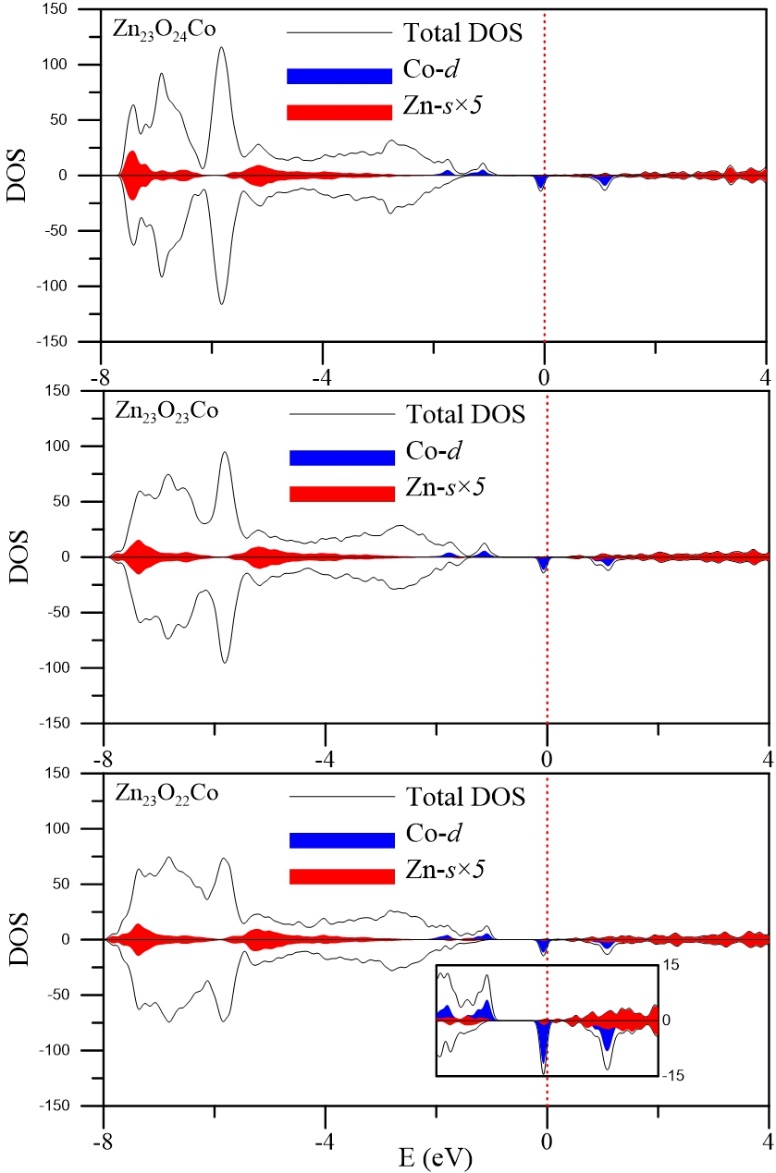


**Figure. S8.** Spin-polarized density of states (DOS) of Co-doped ZnO calculated via DFT for various oxygen vacancy configurations. The total DOS (black), Co 3*d* partial DOS (blue), and Zn 4*s* partial DOS (red, scaled by ×5 for clarity) are plotted for (a) Zn₂₃O₂₄Co (no oxygen vacancy), (b) Zn₂₃O₂₃Co (one oxygen vacancy), and (c) Zn₂₃O₂₂Co (two oxygen vacancies). The inset in (c) highlights the near-Fermi-level region, showing increased spin asymmetry. With increasing oxygen vacancy concentration, the Zn 4*s* states shift toward the Fermi level and exhibit enhanced hybridization with Co 3*d* orbitals. This evolution supports the formation of a vacancy-stabilized impurity band and indicates a carrier-mediated exchange mechanism for the high-T_C_ ferromagnetism in Co-doped ZnO consistent with the RKKY model.

**References**

1. F. de Groot, A. Kotani, *Core Level Spectroscopy of Solids*, CRC Press, Florida **2008**, Ch. 4.
2. M. Kobayashi, Y. Ishida, J. L. Hwang, T. Mizokawa, A. Fujimori, K. Mamiya, J. Okamoto, Y. Takeda, T. Okane, Y. Saitoh, Y. Muramatsu, A. Tanaka, H. Saeki, H. Tabata, T. Kawai, *Phys. Rev. B* **2005**, *72*, 201201.
3. T. Mizokawa, A. Fujimori, *Phys. Rev. B* **1997**, *56*, 6669.
4. G. Kresse, J. Furthmüller, *Phys. Rev. B* **1996**, *54*, 11169.
5. J. P. Perdew, K. Burke, M. Ernzerhof, *Phys. Rev. Lett.* **1996**, *77*, 3865.
6. H. Zhao, X. Zhang, Y. Liu, *Comput. Phys. Commun.* **2023**, *286*, 108664.
7. A. Walsh, J. L. F. Da Silva, S.-H. Wei, *Phys. Rev. Lett.* **2008**, *100*, 256401.
